# Hemoviscoelastography: a new method of hemostasis monitoring

**DOI:** 10.1186/cc12293

**Published:** 2013-03-19

**Authors:** O Tarabrin, S Galich, D Gavrychenko, G Mazurenko, A Suhanov

**Affiliations:** 1 Odessa National Medical University, Odessa, Ukraine

## Introduction

Deep vein thrombosis of lower extremities and pulmonary embolism occupy an important place in the structure of postoperative morbidity and mortality.

## Methods

After ethics approval and informed consent, we studied the functional state of hemostasis in a group of 57 healthy volunteers, who were not receiving drugs affecting coagulation, and 43 patients with postphlebothrombotic syndrome (PPTS). In the PPTS patients we conducted baseline studies of coagulation state and daily monitoring of dynamic changes in the functional state of hemostasis, a comparative evaluation of performance low-frequency piezoelectric vibration hemoviscoelastography (LPVH) and platelet aggregation test (PAT), standard coagulation tests (SCT), and thromboelastogram (TEG).

## Results

We found that LPVH correlated with SCT, PAT and TEG. However, our proposed method is more voluminous: indexes ICC (the intensity of the contact phase of coagulation), t1 (the time for the contact phase of coagulation), and AO (initial rate of aggregation of blood) are consistent with PAT; indexes ICD (the intensity of coagulation drive), CTA (a constant thrombin activity) and CP (the clot intensity of the polymerization) are consistent with SCT and TEG. In addition, the advantage of this method is to determine the intensity of fibrinolysis - with the indicator IRIS (the intensity of the retraction and clot lysis).

## Conclusion

LPVH allows one to make a total assessment of all parts of hemostasis: from initial viscosity and platelet aggregation to coagulation and lysis of clots, as well as their interaction. These figures are objective and informative, as evidenced by close correlation with the performance of traditional coagulation methods.

